# Educational Attainment and Employment Outcome of Survivors of Pediatric CNS Tumors in Switzerland—A Report from the Swiss Childhood Cancer Survivor Study

**DOI:** 10.3390/children9030411

**Published:** 2022-03-14

**Authors:** Maria Otth, Gisela Michel, Nicolas U. Gerber, Ana S. Guerreiro Stücklin, André O. von Bueren, Katrin Scheinemann, on behalf of the Swiss Pediatric Oncology Group (SPOG)

**Affiliations:** 1 Department of Pediatrics, Division of Oncology-Hematology, Kantonsspital Aarau AG, 5001 Aarau, Switzerland; katrin.scheinemann@ksa.ch; 2 Department of Oncology, Hematology, Immunology, Stem Cell Transplantation and Somatic Gene Therapy, University Children’s Hospital Zurich—Eleonore Foundation, 8032 Zurich, Switzerland; nicolas.gerber@kispi.uzh.ch (N.U.G.); ana.stuecklin@kispi.uzh.ch (A.S.G.S.); 3 Department of Health Sciences and Medicine, University of Lucerne, 6002 Lucerne, Switzerland; gisela.michel@unilu.ch; 4 Department of Pediatrics, Obstetrics and Gynecology, Division of Pediatric Hematology and Oncology, University Hospital of Geneva, 1205 Geneva, Switzerland; andre.vonburen@hcuge.ch; 5 Department of Pediatrics, McMaster University, Hamilton, ON L8S 4K1, Canada

**Keywords:** childhood cancer, survivors, education, work, employment, Switzerland

## Abstract

Background: Childhood cancer survivors diagnosed with a central nervous system (CNS) tumor are at risk for educational and vocational challenges. This study compared educational attainment and employment outcome in survivors of CNS tumors to survivors of other malignancies. Methods: The questionnaire-based Swiss Childhood Cancer Survivor Study (SCCSS) included cancer patients diagnosed between 1976 and 2010, aged ≤20 years, who survived ≥5 years after diagnosis. We classified participants aged ≥16 years into three groups: CNS tumor and non-CNS malignancy with and without CNS-directed treatment. We analyzed educational attainment, employment outcome and special schooling. Subgroup analyses included survivors aged ≥25 years. Results: We analyzed 2154 survivors, including 329 (15%) CNS tumor survivors, 850 (40%) non-CNS tumor survivors with and 975 (45%) without CNS-directed treatment. Fewer CNS tumor survivors aged ≥25 years reached tertiary education (44%) compared to those without CNS-directed treatment (51%) but performed similar to survivors with CNS-directed treatment (42%). Among CNS tumor survivors, 36 (14%) received special schooling. Higher parental education was associated with higher levels in survivors. Employment outcome did not significantly differ between the three diagnostic groups. A higher proportion of CNS tumor survivors received disability pension or were unemployed. Conclusions: Our findings suggest that CNS tumor survivors need more time to achieve their highest educational level. This should influence clinical care of these survivors by offering vocational counseling.

## 1. Introduction

Educational attainment and employment outcome are important factors in a person’s life. Educational attainment is determined in childhood and adolescence and has an impact not only on employment status but also on self-confidence, independence, and position in society [1]. A cancer diagnosis and its treatment expose childhood cancer survivors (CCS) to factors that can negatively influence school performance, such as school absences or treatment modalities directed to the central nervous system (CNS), including brain surgery, cranial radiotherapy [2,3,4], or intrathecal chemotherapy [3]. These factors and the primary location of the tumor might cause CNS tumor survivors to face particular difficulties. Previous studies from Denmark, France, Switzerland, the UK, and US compared educational attainment and employment outcome of CCS diagnosed with a CNS tumor to siblings or the general population [2,3,5,6,7]. They found that CNS tumor survivors had lower educational attainment than controls, while survivors diagnosed with non-CNS tumors reached similar educational levels as controls [3,4,5,6,7,8]. None of these studies performed direct comparison of CNS tumor survivors to survivors of other malignancies. We also lack knowledge on the impact of parental education in CNS tumor survivors versus survivors of other malignancies. Higher parental education has a positive impact on a child’s education [9,10]. A previous Swiss study assessed educational attainment in CCS aged 20–40 years at analysis and diagnosed between 1976 and 2003 but did not focus on CNS tumor survivors and did not investigate employment outcome [6]. With this national cohort study, we aimed to close this knowledge gap by describing educational attainment and employment outcome in CNS tumor survivors and comparing them to survivors of other malignancies with or without CNS-directed treatment.

## 2. Materials and Methods

### 2.1. Study Population

The study population consisted of CCS who participated in the Swiss Childhood Cancer Survivor Study (SCCSS), a national questionnaire-based study including all patients registered in the Swiss Childhood Cancer Registry (SCCR), diagnosed between 1976 and 2010, and survived ≥5 years from first cancer diagnosis [11]. The SCCR registers children and adolescents diagnosed with leukemia, lymphoma, CNS tumors, malignant solid tumors, or Langerhans cell histiocytosis since 1976 [12]. Eligible 5-year survivors were asked to participate in the SCCSS between 2007 and 2017 in two waves, where the second wave included the new 5-year survivors. For this study, we included participants aged ≥16 years who have answered at least one question on education or employment outcome. The Ethics Committee of the Canton of Bern approved the SCCR and SCCSS (KEK-BE: 166/2014). The SCCSS is registered at ClinicalTrials.gov (identifier: NCT03297034).

### 2.2. Measurements from the SCCR

Information on demographics, diagnosis and treatment were available from the SCCR, where diagnosis was classified according to the International Classification of Childhood Cancer (ICCC-3) [13]. No WHO classification for CNS tumors was available [14]. Based on the probability of being exposed to CNS-directed treatment, we categorized the CCS into three diagnostic groups: (1) CNS tumors, (2) leukemia and lymphoma, excluding Hodgkin lymphoma as “CNS-directed treatment”, and (3) other malignancies as “without CNS-directed treatment”. We combined leukemia and lymphoma, excluding Hodgkin lymphoma, as their treatment protocols include intrathecal chemotherapy. For chemotherapy, radiotherapy, and surgery we had information on “exposure” and “non-exposure” from the SCCR. Detailed information on radiation field and dose were incomplete and not included in this study.

### 2.3. Measurements from the SCCSS and the Swiss School System

The Swiss school system consists of nine years of compulsory education. After that further education and schooling or vocational education and training can be pursued (Supplementary Figure S1). The questions on educational attainment in the SCCSS were identical to those from the Swiss Health Survey 2007 with nine different school degrees [15]. We condensed these degrees into three categories as recommended by the Swiss conference of cantonal directors of education: primary, secondary, and tertiary education (Supplementary Table S1) [16]. The first SCCSS wave included one question on special schooling. The adult version of the SCCSS included one question on current employment status where we combined the answer options into four categories: employed, not employed, in education, and receiving disability pension (Supplementary Table S2).

We used parental education from the SCCSS as explanatory variable and applied the same categorization into three levels as in CCS. We used the highest educational level achieved by either the mother or the father. Information on language and nationality was also available from the SCCSS.

### 2.4. Statistical Analyses

We used descriptive statistics, including proportion, median, and interquartile range (IQR) to describe the cohort. For comparisons, we used chi-squared tests for categorical variables, rank sum test for continuous variables, and p for trend to test for trends across ordered groups. We took CCS without CNS-directed treatment as reference. We performed subgroup analyses for CCS aged ≥25 years at questionnaire, special schooling, employment outcome, and CNS tumor survivors with and without radiotherapy. We used multivariate regression analysis to assess the association between parental education and CCS’ educational level. For the main analysis, we included CCS aged ≥25 years at questionnaire and performed sensitivity analyses including all CCS. We did not perform analyses stratified by treatment exposures or CNS tumor entities, as we did not have enough detailed information on treatment and diagnosis and the number of survivors per CNS tumor entity were small. We used Stata software package (version 16.0, Stata Corporation, Austin, TX, USA).

## 3. Results

Of 4115 adolescent and adult CCS eligible for the SCCSS, 55% (n = 2245) participated. We excluded 91 participants who did not answer the questions on education or employment, resulting in a final cohort of 2154 CCS. Hereof, 15% (n = 329) were diagnosed with CNS tumors, 40% (n = 850) with leukemia or lymphoma, excluding Hodgkin lymphoma, and 45% (n = 975) with other malignancies (Supplementary Figure S2). Half of participants were male (52%) and the median time after diagnosis was 16 years (Table 1). Sex and main diagnostic category did not differ between participants younger or older than 25 years at questionnaire (Supplementary Table S3). Astrocytoma was the most frequent diagnosis (42%) in CNS tumor survivors. In CCS with CNS-directed treatment, most had leukemia (76%). In those without CNS-directed treatment most had Hodgkin lymphoma (27%) (Supplementary Table S4). CNS tumor survivors had a median age of 10.8 years (IQR 7.0–13.9) at diagnosis and 24.2 years (IQR 20.1–30.4) at questionnaire (Table 1). CNS tumor survivors were older at diagnosis (*p* < 0.001) than survivors with CNS-directed treatment. Compared to survivors with and without CNS directed treatment, CNS tumor survivors had a shorter follow-up (*p* < 0.001 and *p* = 0.008) and were diagnosed in more recent years (*p* < 0.001 and *p* = 0.011). Most CNS tumor survivors had been treated with surgery (96%), which was the only treatment modality in 49%. Chemotherapy was part of the treatment in 28% and radiotherapy in 46%, either alone or in combination with surgery or chemotherapy (Supplementary Table S5).

Most CNS tumor survivors (63%) had reached secondary education as highest educational level, one-fourth (25%) had reached tertiary education and 12% had finished primary education (Table 2). Assuming that some adolescents were still in education, we separately analyzed CCS aged ≥25 years at questionnaire (n = 153) (Table 2). The highest attained educational level in CNS tumor survivors shifted towards a higher proportion of tertiary education (44%), while the proportion of primary and secondary education decreased (10% and 46%). The proportion of survivors who reached tertiary education also increased in both other categories when we analyzed CCS aged ≥25 years (CNS-directed: 32% to 42%; without CNS-directed: 38% to 51%; Table 2). The distribution of the three educational levels differed between CNS tumor survivors and survivors with CNS-directed treatment when taking all age categories into account with more primary education and less tertiary education in CNS tumor survivors (*p* for trend < 0.001). This was no longer significant in CCS aged ≥25 years (p for trend 0.623). In contrast, CNS tumor survivors aged ≥25 years were still less likely to reach higher educational levels compared to survivors without CNS-directed treatment (*p* = 0.035).

We analyzed special schooling in 1852 CCS from the first-wave questionnaire. More CNS tumor survivors had received special schooling compared to both other groups (*p* < 0.001) (Table 2). We analyzed employment outcome in 1692 CCS. Most CNS tumor survivors (82%) were employed or in education, 7% were not employed and 6% received disability pension. In CCS with and without CNS-directed treatment a higher proportion was employed or in education with a lower proportion not employed or receiving disability pension (Table 2).

**Figure 1:**
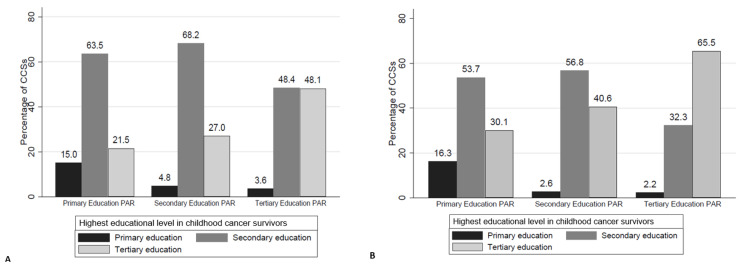
Highest educational level in childhood cancer survivors, represented as bars, stratified by highest parental (PAR) education; (**A**) all survivors (n = 2016), (**B**) survivors ≥25 years (n = 968). Legend: Number of parents per category (**A**): Primary Education PAR n = 200; Secondary Education PAR n = 1113; Tertiary Education PAR n = 703; (**B**): Primary Education PAR n = 123; Secondary Education PAR n = 532; Tertiary Education PAR n = 313; HD = Hodgkin disease/lymphoma, PAR = parents; CCSs = childhood cancer survivors.

**Figure 2:**
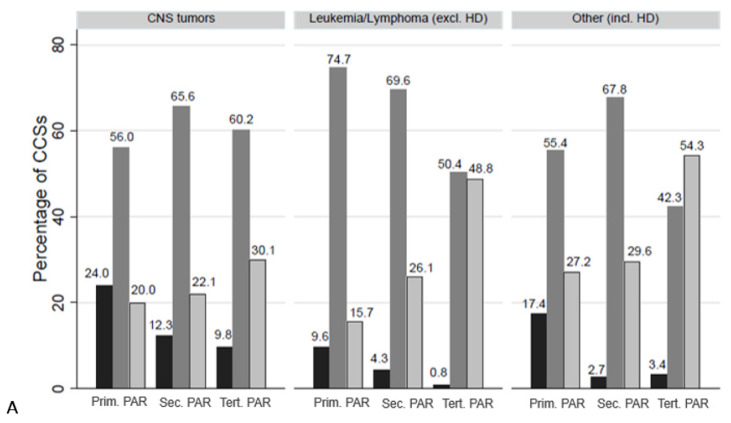

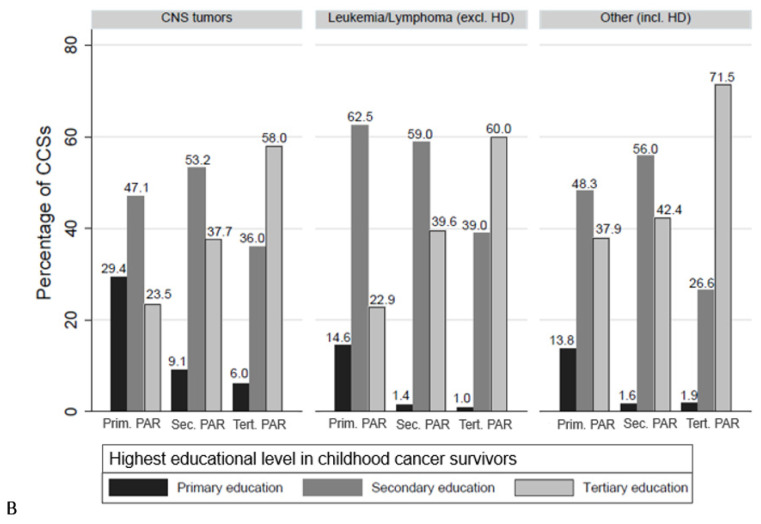
Highest educational level in childhood cancer survivors, represented as bars, stratified by highest parental (PAR) education and by diagnostic group; (**A**) all survivors (n = 2016), (**B**) survivors ≥25 years (n = 968). Abbreviations: HD = Hodgkin disease/lymphoma; Prim. PAR = primary education in parents; Sec. PAR = secondary education in parents; Tert. PAR = tertiary education in parents; HD = Hodgkin disease/lymphoma, PAR = parents; CCSs = childhood cancer survivors.

Stratified by highest parental education, the proportion of CCS reaching primary education decreased and tertiary education increased with increasing parental education, in all CCS, including those aged ≥25 years (Figure 1). This was also true after stratification into the three main diagnostic groups (Figure 2).

The multivariate analysis of all CCS aged ≥25 years showed that children of parents with primary education as highest level were more likely to also reach primary education only (Coefficient, Coeff. 0.084, *p* = 0.005). On the other hand, children of parents with tertiary education were more likely to also reach tertiary education (Coeff. 0.260, *p* < 0.001) (Table 3). A positive coefficient indicates that a certain level of parental education is more probable to be linked to a certain level of survivor education. In CNS tumor survivors, the only significant positive coefficient was between primary education in parents and survivors (Coeff. 0.294, *p* = 0.016). The association was still positive between parental education of secondary and tertiary level and primary education in CNS tumor survivors. These associations were negative in survivors with and without CNS-directed treatment (Table 3). Through all diagnostic categories, the highest educational level of survivors was associated the most positive with parental education of the same level. The associations remained unchanged in the sensitivity analysis, comparing all survivors vs. those aged ≥25 years (Supplementary Table S6). After stratifying CNS tumor survivors by exposure to radiotherapy and by survivors’ educational level, the distribution of parental education did not differ between those exposed and not exposed to radiotherapy (Supplementary Table S7). The same was true for employment.

## 4. Discussion

We found that childhood cancer survivors diagnosed with CNS tumors less frequently reached higher educational levels than CCS with other malignancies. This difference decreased when analyzing CCS aged ≥25 years. Independent of the diagnostic category, survivors’ educational level was associated with the same parental educational level. A higher proportion of CNS tumor survivors had received special schooling and received disability pension compared to survivors of other malignancies. However, the employment outcome did not differ significantly between the diagnostic categories.

CNS tumor survivors represented 15% of all survivors in our cohort. This proportion is similar in the Childhood Cancer Survivor Study (CCSS) with 14% and 13% [3,7] and lies in between the proportion in the French cohort (10%) [2] and the British (21%) [4] and Danish cohort (25%) [5]. Results on educational attainment comparing CCS with siblings or the general population are congruent with our findings. In the Danish cohort, CNS tumor survivors had reduced chances of attaining education at all three levels compared to the general population [5]. The rate ratio for higher education (level 3) was 0.77 (95%CI 0.55–1.07) for male and 0.55 (0.37–0.82) for female CNS tumor survivors. After conditioning on completion of level 2 education, CNS tumor survivors did not differ from controls anymore [5]. This supports our observation that the proportion of CCS reaching tertiary education increased in those aged ≥25 years. In the French cohort, the number of CNS tumor survivors with lower than middle school degree was higher than expected from the general population (observed/expected [95%CI]: 2.3 [1.8–2.9]) [2]. This is similar to our cohort, where 10% of CNS tumor survivors aged ≥25 years had primary education as highest level, compared to 3% and 4% in CCS with and without CNS-directed treatment. In the previous Swiss study, CCS reached tertiary education less frequent than the general population (7.3% vs. 11%) [6]. This difference was no longer significant when only CCS aged ≥27 years were considered (11.3% vs. 14.5%) [6]. We observed the same trend and concluded that CCS need more time to reach higher educational levels than the general population with CNS tumor survivors being particularly affected. Our results show that the distribution of highest educational levels in CNS tumor survivors aged ≥25 years do not differ from those treated with, but from those without CNS-directed treatment. Therefore, not only does the diagnosis of a CNS tumor and its treatment influence education, but CNS-directed treatment influences education as well. Results from the CCSS support this finding, where CCS treated with cranial radiotherapy, intrathecal methotrexate or a combination did significantly more often not complete high school or college compared to siblings [3]. Our results underline the positive effect of parental education on CCS’ educational attainments, similar to other studies [6,17]. More CCS diagnosed with a CNS tumor needed special schooling compared to survivors with and without CNS-directed treatment. In the CCSS, the odds ratio comparing the need of special schooling was higher for survivors of CNS tumors than of other malignancies [3]. For employment outcome, CNS tumor survivors in our cohort showed a trend towards higher proportions of disability pension and not being employed compared to both other groups. In the French cohort, CNS tumor survivors were more often unemployed and seeking work or unemployed because of health than the general population [2]. In a systematic review, CNS tumor survivors were 4.6 times (95%CI 2.56–8.31) more likely to be unemployed than controls [18].

The strengths of this study include the population-based design of the SCCSS, the response rate of 55% for adolescent and adult CCS, and that it is the first study directly comparing educational attainment and employment outcome in CNS tumor survivors to survivors of other types of cancer. Limitations might be linked to changes in the Swiss educational system over time. Through defining three main educational levels, we adapted to these changes, but might still have introduced nondifferential misclassification. As this would have affected all CCS equally, we do not think that this influenced our results. A selection bias might be introduced using questionnaire data. Rueegg et al. could show that nonresponse bias seems to play only a minor role in the SCCSS [19]. However, only 15% of participants were CNS tumor survivors and the median age at diagnosis was higher than expected, which might raise the possibility of non-representativity in this specific group. Information on career aspiration and satisfaction in education and work life were missing. Not every CCS aims for tertiary education. We could not consider this aspect. In addition, we had no information on frequency, reason, and impact of special schooling on education and employment outcome. Based on these limitations we did not perform risk factor analysis stratified by CNS tumor entity or treatment exposure. In addition, detailed information on diagnosis and treatment were not available. The diagnosis of participants over several decades, resulting in different treatment approaches, may additionally affect educational attainment. However, through the stratification in three main diagnostic categories, we took the different underlying diagnoses, treatment strategies, and intensity of CNS-directed treatment into account.

## 5. Conclusions

We conclude that the diagnosis of a CNS tumor in childhood is not necessarily linked to lower educational level, but that CNS tumor survivors might need more time and special support to achieve higher levels. In addition, our results show that CCS exposed to CNS-directed treatment might also benefit from special educational support. This important information should be considered in long-term follow-up care and vocational counseling of CCS.

## Figures and Tables

**Table 1:** Demographic and clinical characteristics of included adolescent and adult childhood cancer survivors; N = 2154.

	CNS Tumors (n = 329)	*p*-Value ^1^ (CNS vs. No CNS-Directed)	CNS-Directed Treatment ^2^ (n = 850)	*p*-Value ^1^ (CNS vs. CNS-Directed)	*p*-Value ^1^ (CNS-directed vs. No CNS-Directed)	without CNS-Directed Treatment ^3^ (n = 975)	Total (n = 2154)
	n (%)						
**Sociodemographic characteristics**							
**Gender**		0.055		0.417	**<0.001**		
Male	176 (54)		477 (56)			462 (47)	1115 (52)
**Language**		0.917		0.080	**0.011**		
German	217 (66)		605 (71)			640 (66)	1462 (68)
Other than German	112 (34)		245 (28)			335 (34)	692 (32)
**Nationality**		0.954		0.409	0.278		
**Swiss**	302 (92)		767 (90)			894 (92)	1963 (91)
Other than Swiss	27 (8)		83 (10)			81 (8)	191 (9)
**Age at diagnosis**, median years (IQR)	10.8 (7.0–13.9)	0.176	7.4 (3.8–12.2)	**<0.001**	**<0.001**	11.9 (4.8–15.7)	10.0 (4.5–14.3)
<5	46 (14)		299 (35)			251 (26)	596 (28)
5–9	98 (30)		224 (27)			152 (16)	474 (22)
10–14	129 (39)		231 (27)			276 (28)	636 (29)
15–21	56 (17)		96 (11)			296 (30)	448 (21)
**Age at survey**, median years (IQR)	24.2 (20.1–30.4)	**0.027**	24.2 (19.9–30.6)	0.938	**0.002**	25.3 (20.5–33.1)	24.6 (20.1–31.4)
15–24	176 (54)		456 (54)			478 (49)	1110 (52)
25–34	100 (30)		280 (33)			304 (31)	684 (32)
≥35	53 (16)		114 (13)			193 (20)	360 (16)
**Follow-up time**, median years (IQR)	13.9 (9.1–20.5)	**0.008**	16.5 (11.8–22.3)	**<0.001**	**0.016**	16.1 (10.2–21.9)	16.1 (10.6–21.9)
5–14	181 (55)		353 (42)			429 (44)	963 (45)
15–24	107 (33)		351 (41)			397 (41)	855 (40)
≥25	41 (12)		146 (17)			149 (15)	336 (15)
**Year of diagnosis**		**0.011**		**<0.001**	**<0.001**		
1970–1989	88 (27)		354 (42)			343 (35)	785 (37)
1990–1999	137 (42)		346 (41)			384 (39)	867 (40)
2000–2010	104 (31)		150 (17)			248 (26)	502 (23)

^1^ *p*-value as chi squared test for categorical variables and rank sum test for continuous variables. ^2^ Leukemia and lymphoma, excluding Hodgkin lymphoma. ^3^ Other tumors, including Hodgkin lymphoma, serve as reference. Significant *p*-values are highlighted in bold.

**Table 2:** Educational attainment, special schooling, and current employment status in adult and adolescent childhood cancer survivors; N = 2154.

	CNS Tumors	*p* for Trend ^1^ (No CNS- Directed vs. CNS)	CNS-Directed Treatment ^2^	*p* for Trend ^1^ (No CNS-Directed vs. CNS directed)	*p* for Trend ^1^ (CNS-Directed vs. CNS)	without CNS-Directed Treatment ^3^	Total
**Characteristics**	n (%)						
**Three educational levels in all survivors**	(n = 329)	**<0.001**	(n = 850)	**0.016**	**<0.001**	(n = 975)	(n = 2154)
Primary Education	38 (12)		36 (4)			44 (5)	118 (5)
Secondary Education	208 (63)		542 (64)			560 (57)	1310 (61)
Tertiary Education	83 (25)		272 (32)			371 (38)	726 (34)
**Three educational levels in survivors ≥25 years**	(n = 153)	**0.035**	(n = 394)	**0.018**	0.623	(n = 497)	(n = 1044)
Primary Education	15 (10)		13 (3)			19 (4)	47 (4)
Secondary Education	71 (46)		214 (54)			224 (45)	509 (49)
Tertiary Education	67 (44)		167 (42)			254 (51)	488 (47)
Age at survey (mean, IQR)	30.8 (27.5–36.3)		30.9 (27.6–35.9)			32.8 (28.2–38.3)	31.8 (27.9–37.1)
**Special schooling**	(n = 261)	**<0.001**	(n = 766)	0.071	**<0.001**	(n = 825)	(n = 1852)
Yes	36 (14)		22 (3)			38 (5)	96 (5)
No	219 (84)		721 (94)			764 (93)	1704 (92)
Missing	6 (2)		23 (3)			23 (3)	52 (3)
**Employment outcome**	(n = 251)	0.141	(n = 660)	0.202	0.595	(n = 781)	(n = 1692)
Employed	162 (65)		447 (68)			553 (71)	1162 (69)
Not employed	18 (7)		34 (5)			30 (4)	82 (5)
In education	42 (17)		148 (22)			158 (20)	348 (21)
Disability pension	15 (6)		12 (2)			15 (2)	42 (2)
Missing	14 (5)		19 (3)			25 (3)	58 (3)

^1^ *p* for trend (nptrend in STATA); ^2^ Leukemia and lymphoma, excluding Hodgkin lymphoma; ^3^ Other tumors, including Hodgkin lymphoma. Significant *p*-values are highlighted in bold.

**Table 3:** Multivariate regression analysis evaluating the association between highest parental educational level and highest educational level achieved in childhood cancer survivors aged ≥25 years at survey, stratified by diagnostic group.

	Coefficient	95%CI	*p*-Value
**All survivors aged ≥25 years (n = 1044)**			
**Primary education CCS**			
Primary education parents	0.084	0.025–0.141	0.005
Secondary education parents	−0.053	−0.101–−0.004	0.035
Tertiary education parents	−0.056	−0.107–−0.006	0.029
**Secondary education CCS**			
Primary education parents	0.010	−0.129–0.150	0.886
Secondary education parents	0.041	−0.076–0.159	0.490
Tertiary education parents	−0.204	−0.327–−0.081	0.001
**Tertiary education CCS**			
Primary education parents	−0.094	−0.232–0.044	0.183
Secondary education parents	0.011	−0.105–0.127	0.849
Tertiary education parents	0.260	0.139–0.382	<0.001
**CNS tumor survivors (n = 153)**			
**Primary education CCS**			
Primary education parents	0.294	0.056–0.532	0.016
Secondary education parents	0.090	−0.112–0.294	0.378
Tertiary education parents	0.06	−0.149–0.268	0.571
**Secondary education CCS**			
Primary education parents	0.026	−0.380–0.433	0.899
Secondary education parents	0.088	−0.259–0.435	0.618
Tertiary education parents	−0.084	−0.442–0.272	0.641
**Tertiary education CCS**			
Primary education parents	−0.320	−0.718–0.077	0.114
Secondary education parents	−0.179	−0.519–0.161	0.300
Tertiary education parents	0.024	−0.325–0.374	0.890
**Survivors with CNS-directed treatment (n = 394)**			
**Primary education CCS**			
Primary education parents	0.077	−0.003–0.157	0.061
Secondary education parents	−0.055	−0.122–0.013	0.112
Tertiary education parents	−0.059	−0.131–0.012	0.104
**Secondary education CCS**			
Primary education parents	0.004	−0.223–0.232	0.970
Secondary education parents	−0.031	−0.222–0.160	0.750
Tertiary education parents	−0.230	−0.433–−0.027	0.026
**Tertiary education CCS**			
Primary education parents	−0.081	−0.304–0.141	0.474
Secondary education parents	0.086	−0.102–0.274	0.369
Tertiary education parents	0.289	0.091–0.488	0.004
**Survivors without CNS-directed treatment (n = 497)**			
**Primary education CCS**			
Primary education parents	0.033	−0.044–0.109	0.405
Secondary education parents	−0.088	−0.153–−0.024	0.007
Tertiary education parents	−0.086	−0.153–−0.019	0.011
**Secondary education CCS**			
Primary education parents	0.009	−0.188–0.207	0.928
Secondary education parents	0.086	−0.079–0.251	0.307
Tertiary education parents	−0.208	−0.379–−0.037	0.017
**Tertiary education CCS**			
Primary education parents	−0.042	−0.239–0.1558	0.678
Secondary education parents	0.003	−0.162–0.168	0.973
Tertiary education parents	0.294	0.123–0.465	0.001

Abbreviations: CCS, childhood cancer survivor; CNS, central nervous system.

## Data Availability

The datasets analyzed during the current study are available from the corresponding author on reasonable request.

## Supplementary Materials

The following supporting information can be downloaded at: https://www.mdpi.com/article/10.3390/children9030411/s1, Table S1: Question from the SCCSS on vocational training and its categorization according to the Swiss education system and the respective combinations used in this study; Table S2: Question from the SCCSS on current employment situation and the respective combinations used in this study; Table S3: Characteristics of adult and adolescent childhood cancer survivors younger and older than 25 years at survey, N = 2154; Table S4: Specification of diagnostic groups according to ICCC3 categorization; Table S5: Treatment characteristics for first diagnosis of adult and adolescent childhood cancer survivors, including only those with treatment data available, N = 2085; Table S6: Multivariate regression analysis evaluating the association between highest parental educational level and highest educational level achieved in all childhood cancer survivors and in those aged ≥25 years at survey; Table S7: Characteristics of childhood cancer survivors diagnosed with a CNS tumor, stratified by exposure to radiotherapy (n = 310); Figure S1: Educational levels in the Swiss education system and its explanation, adapted from the Swiss conference of cantonal directors of education; Figure S2: Patient tree. Participants framed with solid line (n = 2154) correspond to whole population aged ≥16 years. Participants framed with dashed line (n = 1044) correspond to subpopulation aged ≥25 years.

## References

[1] Galobardes B., Shaw M., Lawlor D.A., Lynch J.W., Davey Smith G. (2006). Indicators of socioeconomic position (part 1). J. Epidemiol. Community Health.

[2] Dumas A., Berger C., Auquier P., Michel G., Fresneau B., Allodji R.S., Haddy N., Rubino C., Vassal G., Valteau-Couanet D. (2016). Educational and occupational outcomes of childhood cancer survivors 30 years after diagnosis: A French cohort study. Br. J. Cancer.

[3] Mitby P.A., Robison L.L., Whitton J.A., Zevon M.A., Gibbs I.C., Tersak J.M., Meadows A.T., Stovall M., Zeltzer L.K., Mertens A.C. (2003). Utilization of special education services and educational attainment among long-term survivors of childhood cancer: A report from the Childhood Cancer Survivor Study. Cancer.

[4] Lancashire E.R., Frobisher C., Reulen R.C., Winter D.L., Glaser A., Hawkins M.M. (2010). Educational attainment among adult survivors of childhood cancer in Great Britain: A population-based cohort study. J. Natl. Cancer Inst..

[5] Koch S.V., Kejs A.M., Engholm G., Johansen C., Schmiegelow K. (2004). Educational attainment among survivors of childhood cancer: A population-based cohort study in Denmark. Br. J. Cancer.

[6] Kuehni C.E., Strippoli M.P., Rueegg C.S., Rebholz C.E., Bergstraesser E., Grotzer M., von der Weid N.X., Michel G. (2012). Educational achievement in Swiss childhood cancer survivors compared with the general population. Cancer.

[7] Armstrong G.T., Liu Q., Yasui Y., Huang S., Ness K.K., Leisenring W., Hudson M.M., Donaldson S.S., King A.A., Stovall M. (2009). Long-term outcomes among adult survivors of childhood central nervous system malignancies in the Childhood Cancer Survivor Study. J. Natl. Cancer Inst..

[8] Boman K.K., Lindblad F., Hjern A. (2010). Long-term outcomes of childhood cancer survivors in Sweden: A population-based study of education, employment, and income. Cancer.

[9] Dubow E.F., Boxer P., Huesmann L.R. (2009). Long-term effects of parents’ education on children’s educational and occupational success: Mediation by family interactions, child aggression, and teenage aspirations. Merrill-Palmer Quarterly.

[10] Spera C., Wentzel K.R., Matto H.C. (2009). Parental aspirations for their children’s educational attainment: Relations to ethnicity, parental education, children’s academic performance, and parental perceptions of school climate. J. Youth Adolesc..

[11] Kuehni C.E., Rueegg C.S., Michel G., Rebholz C.E., Strippoli M.P., Niggli F.K., Egger M., von der Weid N.X. (2012). Cohort profile: The Swiss childhood cancer survivor study. Int. J. Epidemiol..

[12] Michel G., von der Weid N.X., Zwahlen M., Adam M., Rebholz C.E., Kuehni C.E. (2007). The Swiss childhood cancer registry: Rationale, organisation and results for the years 2001–2005. Swiss Med. Wkly..

[13] Steliarova-Foucher E., Stiller C., Lacour B., Kaatsch P. (2005). International classification of childhood cancer. Cancer.

[14] Louis D.N., Perry A., Reifenberger G., von Deimling A., Figarella-Branger D., Cavenee W.K., Ohgaki H., Wiestler O.D., Kleihues P., Ellison D.W. (2016). The 2016 World Health Organization classification of tumors of the central nervous system: A summary. Acta Neuropathol..

[15] Bundesamt für Statistik (2008). Schweizerische Gesundheitsbefragung 2007, Erste Ergebnisse.

[16] Schweizerische Konferenz der Kantonalen Erziehungsdirektoren The Swiss Education System 2020. https://www.edk.ch/dyn/16342.php.

[17] Maule M., Zugna D., Migliore E., Alessi D., Merletti F., Onorati R., Zengarini N., Costa G., Spadea T. (2017). Surviving a childhood cancer: Impact on education and employment. Eur. J. Cancer Prev..

[18] Mader L., Michel G., Roser K. (2017). Unemployment following childhood cancer. Dtsch. Arztebl. Int..

[19] Rueegg C.S., Gianinazzi M.E., Michel G., Zwahlen M., von der Weid N.X., Kuehni C.E. (2017). No evidence of response bias in a population-based childhood cancer survivor questionnaire survey—Results from the Swiss childhood cancer survivor study. PLoS ONE.

