# Supplementary material for: Educational Attainment and Employment Outcome of Survivors of Pediatric CNS Tumors in Switzerland—A Report from the Swiss Childhood Cancer Survivor Study
Source: Children (Basel). 2022 Mar 14;9(3):411. doi: 10.3390/children9030411 (PMC8947698; doi:10.3390/children9030411)
Supplement: Supplementary file 1 [file children-09-00411-s001.zip › children-1573207-supplementary.pdf]

Supplementary Material

Educational Attainment and Employment Outcome of Survivors of Pediatric CNS Tumors in Switzerland—A Report from the Swiss Childhood Cancer Survivor Study

Children

Maria Otth, Gisela Michel, Nicolas Gerber, Ana S. Guerreiro Stücklin, André von Bueren, Katrin Scheinemann, for the Swiss Pediatric Oncology Group (SPOG)

Corresponding author
Maria Otth, MD
Division of Oncology-Hematology, Department of Pediatrics
Kantonsspital Aarau
Tellstrasse, 5001 Aarau, Switzerland
E-mail: maria.otth@ksa.ch

Supplemental Table S1: Question from the SCCSS on vocational training and its categorization according to the Swiss education system and the respective combinations used in this study (11, 15)

Q: Questions about your **vocational training**. Enter all your completed training in the column a) and your current training in column b).
Several answers are possible.

	**a) Completed training**	**b) Current training**	**Five educational categories**	**Three educational levels**
Compulsory school (primary school, secondary school, orientation school, pre-high school, special school)	☐	☐	Compulsory school	**Primary education**
"Diplommittelschule" (up to 2 years), traffic school, social year, preliminary course for nursing professions (1 or 2 years), school preparing for apprenticeship	☐	☐	Vocational education and training	**Secondary education**
Apprenticeship or full-time vocational school (e.g. commercial school)	☐	☐		
High school, "Diplommittelschule" (3 years)	☐	☐	High school, teacher training	
Teacher seminars (e.g. kindergarten, elementary school), Music teacher, gymnastics and sports teachers	☐	☐		
Vocational training, technical training (e.g. federal certificate, federal certificate of specialized knowledge, federal diploma), technical or master diploma, higher commercial comprehensive school, technical school	☐	☐	Diploma, technical schools	
Higher technical school (e.g. HTL, HWV, HFG, HFS) for full-time training with a minimum duration of 3 years (including postgraduate diploma)	☐	☐		
University of applied sciences (including postgraduate diploma)	☐	☐	Higher vocational, university	**Tertiary education**
University, college (including post-graduate studies)	☐	☐		

Supplemental Table S2: Question from the SCCSS on current employment situation and the respective combinations used in this study

Q: Current employment situation. *Indicate all applicable answers, more than one answer is possible*

	Number of hours per week	Combination of answers for this study
☐ Employment (fulltime)		Employed
☐ Employment (part-time)		
☐ Multiple employments (part-time)		
☐ Unemployed		Not employed
☐ Not employed, on job search		
☐ Not employed, future employment assured		
☐ Not employed, not on job search		
☐ In education (school, study, apprenticeship)		In education
☐ Pension recipient		Disability pension

Supplemental Table S3: Characteristics of adult and adolescent childhood cancer survivors younger and older than 25 years at survey, N=2154

	**Aged <25 years** (n=1,110)	**Aged ≥25 years** (n=1044)	**p-value**[1]	**Total** (n=2,154)
	n(%)	n(%)		n(%)
**Gender**				
Male	563 (51)	552 (53)	0.318	1,115 (52)
**Language**				
German	757 (68)	705 (67)	0.739	1,462 (68)
**Nationality**				
Swiss	1003 (90)	960 (92)	0.193	1,963 (91)
**Diagnostic categories**				
CNS tumors	176 (16)	153 (15)	0.106	329 (15)
CNS-directed treatment[2]	456 (41)	394 (38)		850 (40)
No CNS-directed treatment[3]	478 (43)	497 (47)		975 (45)
**CNS tumor groups according to ICCC3**				
IIIa: Ependymoma and choroid plexus tumors	11(6)	15 (10)	0.325	26 (8)
IIIb: Astrocytoma	73 (41)	64 (42)		137 (42)
IIIc: Intracranial and intraspinal embryonal tumors	36 (20)	20 (13)		56 (17)
IIId: Other gliomas: 20	10 (6)	10 (6)		20 (6)
IIIe: Other specified intracranial /-spinal neoplasms	37 (21)	30 (20)		67 (20)
IIIf: Unspecified intracranial /-spinal neoplasms	1 (1)	4 (3)		5 (2)
Xa: Intracranial /-spinal germ cell tumors	8 (5)	10 (6)		18 (5)
**Age at diagnosis**, median years (IQR)	7.4 (3.3 – 12.2)	12.5 (6.5 – 16.3)	<0.001	10.0 (4.5 – 14.3)
<5	404 (36)	192 (18)		596 (28)
5-9	273 (25)	201 (19)		474 (22)
10-14	338 (30)	298 (29)		636 (29)
15-21	95 (9)	353 (34)		448 (21)
**Age at survey**, median years (IQR)	20.2 (18.1 – 22.2)	31.8 (27.9 – 37.1)	<0.001	24.6 (20.1-31.4)
**Follow-up time**, median years (IQR)	11.9 (8.1 – 16.0)	21.9 (16.5 – 26.6)	<0.001	16.1 (10.6-21.9)
**Year of diagnosis**				
1970 – 1989	147 (12)	638 (61)	<0.001	785 (37)
1990 - 1999	570 (51)	297 (28)		867 (40)
2000 - 2010	393 (35)	109 (11)		502 (23)

[1] p-value as chi squared test for categorical variables and rank sum test for continuous variables
[2] Leukemia and lymphoma, excluding Hodgkin lymphoma
[3] Other tumors, including Hodgkin lymphoma, serve as reference

Supplemental Table S4: Specification of diagnostic groups according to ICCC3 categorization

**CNS tumors**<br>n=329	**CNS-directed treatment**[1]<br>n=850	**No CNS-directed treatment**[2]<br>n=975
n(%)	n(%)	n(%)
III: CNS tumors: 311 (94%)<br>IIIa: Ependymoma and choroidplexus tumors: 26<br>IIIb: Astrocytoma: 137<br>IIIc: Intracranial /-spinal embryonal tumors: 56<br>IIId: Other gliomas: 20<br>IIIe: Other specified intracranial /-spinal neoplasms; 67<br>IIIf: Unspecified intracranial /-spinal neoplasms: 5<br>Xa: Intracranial /-spinal germ cell tumors: 18 (6%)	I: Leukemia 647 (76%)<br>IIb-e: Lymphoma 203 (24%)	IIa: Lymphoma: 268 (27%)<br>IV: Neuroblastoma: 74 (8%)<br>V: Retinoblastoma: 39 (4%)<br>VI: Renal tumors: 109 (11%)<br>VII: Hepatic tumors: 12 (1%)<br>VIII: Malignant bone tumors: 115 (12%)<br>IX: Soft tissue: 132 (14%)<br>X: Germ cell tumors: 97 (9%)<br>XI: Other malignant epithelial tumors: 57 (6%)<br>Other: 4 (1%)<br>LCH: 68 (7%)

[1] Leukemia and lymphoma, excluding Hodgkin lymphoma
[2] Other tumors, including Hodgkin lymphoma

Supplemental Table S5: Treatment characteristics for first diagnosis of adult and adolescent childhood cancer survivors, including only those with treatment data available, N=2085

	**CNS tumors** (n=310)	**p-value**[1] (CNS vs. No CNS-directed)	**CNS-directed treatment**[2] (n=841)	**p-value**[1] (CNS vs. CNS-directed)	**p-value**[1] (CNS-directed vs. No CNS-directed)	**No CNS-directed treatment**[3] (n=934)	**Total** (n=2,085)
**Treatment**	n (%)						
Surgery yes	299 (96)		432 (51)			907 (97)	1638 (79)
Chemotherapy yes	89 (28)		833 (99)			733 (78)	1655 (79)
Radiotherapy yes	142 (46)		241 (28)			403 (43)	786 (38)
HSCT	6 (2)		74 (9)			31 (3)	111 (5)
**Treatment combined**							
Surgery Only		<**0.001**		<**0.001**	<**0.001**		
Yes	151 (49)		1 (1)			150 (16)	302 (14)
No	159 (51)		840 (99)			784 (84)	1783 (86)
Chemotherapy[4]		<**0.001**		<**0.001**	<**0.001**		
Yes	10 (3)		592 (70)			377 (40)	979 (47)
No	300 (97)		249 (30)			557 (60)	1106 (53)
Radiotherapy[5]		0.414		<**0.001**	<**0.001**		
Yes	142 (46)		241 (28)			403 (43)	786 (38)
No	168 (54)		600 (72)			531 (57)	1299 (62)

[1] p-value as chi squared test for categorical variables
[2] Leukemia and lymphoma, excluding Hodgkin lymphoma
[3] Other tumors, including Hodgkin lymphoma
[4] Chemotherapy without radiotherapy, may have had surgery
[5] Radiotherapy, may have had surgery and/or chemotherapy
[6] Hematopoietic stem cell transplantation, including autologous and allogeneic, may have had surgery and/or chemotherapy and/or radiotherapy

TABLE S6: Multivariate regression analysis evaluating the association between highest parental educational level and highest educational level achieved in all childhood cancer survivors and in those aged ≥25 years at survey

	Coefficient	95%CI	p-value
**All survivors (n=2,154)**			
**Primary education CCS**			
Primary education parents	0.077	0.286 – 0.125	**0.022**
Secondary education parents	-0.025	-0.065 – 0.015	0.222
Tertiary education parents	-0.037	-0.078 – 0.004	0.079
**Secondary education CCS**			
Primary education parents	0.026	-0.078 – 0.131	0.621
Secondary education parents	0.073	-0.012 – 0.158	0.091
Tertiary education parents	-0.125	-0.213 – -0.037	**0.005**
**Tertiary education CCS**			
Primary education parents	-0.104	-0.204 – -0.004	0.042
Secondary education parents	-0.048	-0.130 – 0.033	0.246
Tertiary education parents	0.162	0.077 – 0.246	**<0.001**
**Survivors aged ≥25 years (n=1044)**			
**Primary education CCS**			
Primary education parents	0.084	0.025 – 0.141	**0.005**
Secondary education parents	-0.053	-0.101 – -0.004	**0.035**
Tertiary education parents	-0.056	-0.107 – -0.006	**0.029**
**Secondary education CCS**			
Primary education parents	0.010	-0.129 – 0.150	0.886
Secondary education parents	0.041	-0.076 – 0.159	0.490
Tertiary education parents	-0.204	-0.327 – -0.081	**0.001**
**Tertiary education CCS**			
Primary education parents	-0.094	-0.232 – 0.044	0.183
Secondary education parents	0.011	-0.105 – 0.127	0.849
Tertiary education parents	0.260	0.139 – 0.382	**<0.001**

TABLE S7: Characteristics of childhood cancer survivors diagnosed with a CNS tumor, stratified by exposure to radiotherapy (n=329)

	Radiotherapy YES	Radiotherapy NO	Radiotherapy Missing	p-value[1]	Total
	n(%)	n(%)	n(%)		n(%)
**Demographic characteristics**					
**Population (all CNS tumor survivors)**	n=142	n=168	n=19		n=329
**Gender**					
Male	77	89	10	0.973	176
**Age at diagnosis, median [IQR]**	9.9 [6.8 – 13.3]	11.5 [7.0 – 14.3]	11.4 [8.6 – 15.1]	0.095	10.8 [7.0 – 13.9]
**CNS tumor groups according to ICCC3**					
IIIa: Ependymoma and choroid plexus tumors	17 (65)	8 (31)	1 (4)		26 (100)
IIIb: Astrocytoma	31 (23)	94 (68)	12 (9)		137 (100)
IIIc: Intracranial and intraspinal embryonal tumors	55 (98)	1 (2)	0		56 (100)
IIId: Other gliomas	6 (30)	13 (65)	1 (5)		20 (100)
IIIe: Other specified intracranial /-spinal	17 (25)	46 (69)	4 (6)		67 (100)
neoplasms	1 (20)	4 (80)	0		5 (100)
IIIf: Unspecified intracranial /-spinal neoplasms	15 (83)	2 (11)	1 (6)		18 (100)
Xa: Intracranial /-spinal germ cell tumors					
**Educational levels**					
**Population (all CNS tumor survivors)**	n=142	n=168	n=19		n=329
**Primary education CCS**	**22**	**13**	**3**	0.652	**38**
Primary education parents	4 (18)	2 (15)	0		6 (16)
Secondary education parents	11 (50)	8 (62)	1		20 (53)
Tertiary education parents	7 (32)	3 (23)	2		12 (31)
Parental education missing	0	0			0
**Secondary education CCS**	**97**	**104**	**7**	0.533	**208**
Primary education parents	9 (9)	4 (4)			14 (7)
Secondary education parents	49 (51)	54 (52)			107 (51)
Tertiary education parents	35 (36)	37 (35)			74 (36)
Parental education missing	4 (4)	9 (9)			13 (6)
**Tertiary education CCS**	**23**	**51**	**9**	0.828	**83**
Primary education parents	2 (9)	3 (6)			5 (6)
Secondary education parents	10 (43)	21 (41)			36 (43)
Tertiary education parents	9 (39)	25 (49)			37 (45)
Parental education missing	2 (9)	2 (4)			5 (6)

**Employment outcome**					
**Population (participants in adult questionnaire)**	n=110	n=123	n=18		n=251
Employed	73 (66)	79 (64)	10 (55)	0.514	162 (64)
Not employed	4 (4)	12 (10)	2 (11)		18 (8)
In education	17 (15)	22 (18)	3 (17)		42 (17)
Early retirement	9 (8)	5 (4)	1 (6)		15 (6)
Missing	7 (6)	5 (4)	2 (11)		14 (5)

[1] p-value: comparing CNS tumor survivors with and without radiotherapy; chi squared test for categorical variables, ranksum for continuous variables

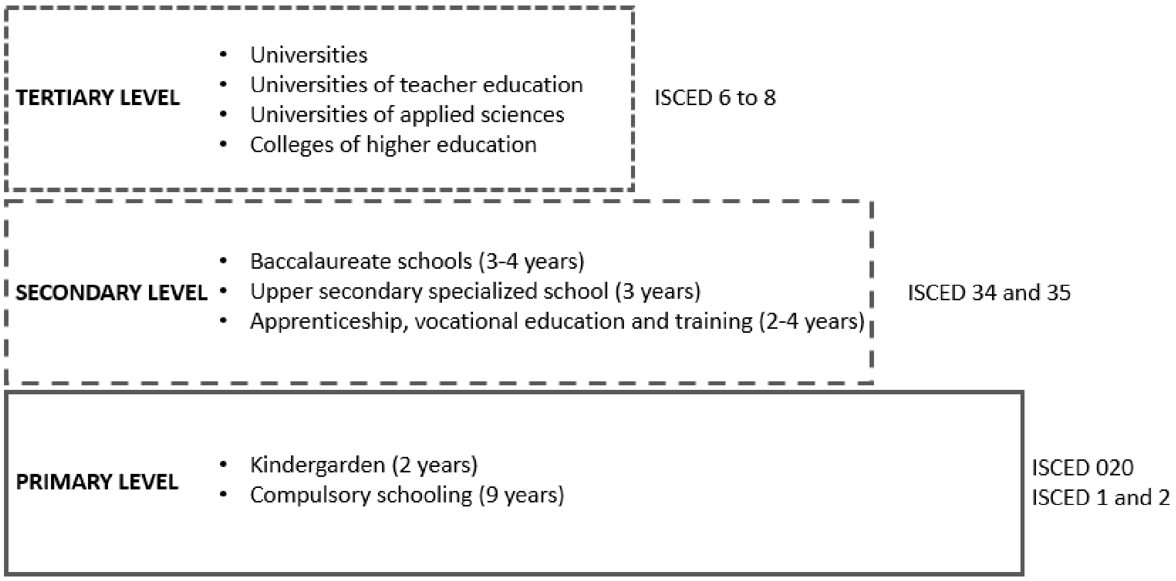

ISCED, International Standard Classification of Education 2011

Supplemental Figure S1: Educational levels in the Swiss education system and its explanation, adapted from the Swiss conference of cantonal directors of education (16)

Short description of some of the educational paths in the secondary levels in more detail:

- Swiss residents who finish primary educational level only with no additional education: often work as assistant worker or construction worker; no work with a lot of responsibilities
- Vocational education and training (VET): tailored to >250 different professions. VET consists of two parts: 3-4 days per week practical training (apprenticeship) and 1-2 days per week theoretical classes (vocational and general educational subjects). Finishing VET leads to professions such as pharma assistant, carpenter, electrician, secretary, nurse, salesperson
- Upper secondary specialized school: provide general education at upper secondary level and offer preparation for tertiary level professional education. Alternative to Baccalaureate school
- Baccalaureate school: does not lead to professional qualifications; prepares for tertiary level education programs

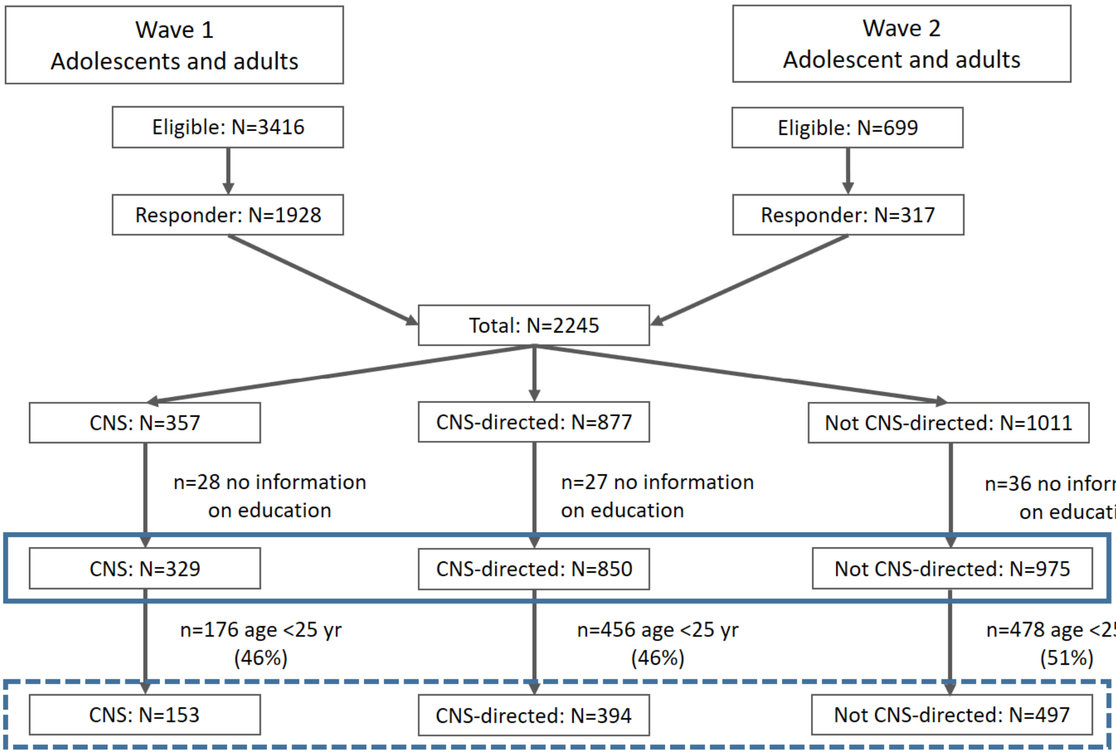

Supplemental Figure S2: Patient tree. Participants framed with solid line (n=2154) correspond to whole population aged ≥16 years. Participants framed with dashed line (n=1044) correspond to sub-population aged ≥25 years.
